# Premature Fatigue Failure Analysis of Axle in Permanent Magnet Direct-Drive Electric Locomotive

**DOI:** 10.3390/ma18163747

**Published:** 2025-08-11

**Authors:** An-Xia Pan, Chao Wen, Haoyu Wang, Peng Shi, Quanchang Bi, Xicheng Jia, Ping Tao, Xuedong Liu, Yi Gong, Zhen-Guo Yang

**Affiliations:** 1 School of Mechanical Engineering and Rail Transit, Changzhou University, Changzhou 213164, China; pax508@cczu.edu.cn (A.-X.P.); lxd@cczu.edu.cn (X.L.); 2 Jiangsu Key Laboratory of Green Process Equipment, Changzhou University, Changzhou 213164, China; 3 Institute of Materials Modification and Modelling, School of Materials Science and Engineering, Shanghai Jiao Tong University, Shanghai 200240, China; 4 Shanghai Key Laboratory of Materials Laser Processing and Modification, Shanghai Jiao Tong University, Shanghai 200240, China; 5 CRRC Qishuyan Institute Co., Ltd., Changzhou 213011, China; 6 Department of Materials Science, Fudan University, Shanghai 200433, China

**Keywords:** axle, permanent magnet locomotive, fatigue fracture, failure analysis, finite element analysis

## Abstract

This study investigates premature fatigue failures in three EA1N steel axles from permanent magnet direct-drive locomotives during wheel-seat bending tests. Complete fracture occurred in one axle at 3 million cycles, and in the other two axles, cracks appeared and were observed through magnetic particle detection at 3.5 million and 1.6 million cycles, respectively. A comprehensive failure analysis was conducted through metallurgical examination, fractography, mechanical testing, residual stress measurement, and finite element analysis. The fractographic results revealed fractures consistently initiated at the wheel-seat to axle-body transition arc, exhibiting characteristic ratchet marks and beach patterns. The premature fracture mechanism was identified as a high-stress fatigue fracture. The residual stress measurements showed detrimental tensile stresses at the surface. Coupled with the operating stress, the stress on the axle exceeds fatigue strength, which accelerates the initiation and propagation of fatigue cracks. Based on these observations, the failure mechanism was identified, and preventive methods were proposed to reduce the risk of recurrence of the in-service axles.

## 1. Introduction

Rail transit systems are critical infrastructure components supporting modern societal development and transport networks. The wheel–axle assembly includes interference fit and rigid connections, which are subjected to complex dynamic load conditions during train operation. This operating state not only puts forward requirements on the chemical composition and mechanical properties of the axle material but also imposes high requirements on fatigue performance [1,2,3]. Research on axle reliability and damage over the years has found that, in addition to wear and tear, about two-thirds of axle damage is caused by fatigue. Under cyclic loading conditions typical of rail operations, localized plastic deformation accumulates at stress concentration zones, initiating microstructural damage that progressively develops into macroscopic cracks. This damage evolution follows characteristic stages: (i) dislocation accumulation forming persistent slip bands, (ii) microcrack nucleation at grain boundaries or inclusions, and (iii) stable crack propagation until critical fracture occurs [4,5,6]. The fatigue process is particularly sensitive to microstructure, surface conditions, residual stresses, non-metallic inclusions, and stress concentration factors at geometric transitions [7,8,9], which collectively determine the component’s service life.

**Figure 1:**
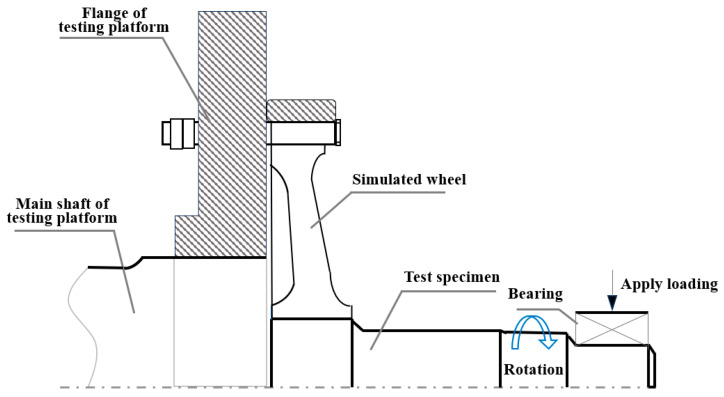
Schematic diagram of permanent magnet direct-drive wheelset.

The permanent magnet direct-drive (PMDD) electric locomotive is characterized by its bogie structure using permanent magnet motor technology, wherein the motor is directly mounted on the axle to achieve drive transmission, eliminating the need for gearboxes and couplings. This design not only reduces the weight of the bogie itself but also prevents transmission noise, oil leakage risks, and transmission losses of the gearbox [10]. These benefits have positioned PMDD as the preferred solution for both heavy-haul locomotives and urban transit systems, where reliability and energy efficiency are paramount. However, compared to conventional bogie axles, the axle in PMDD systems faces more stringent mechanical demands. In traditional bogies, the motor and gearbox are mounted on the bogie frame, and the axle mainly carries part of the load from the gearbox. In contrast, the PMDD motors are directly integrated with the axle (as shown in Figure 1), requiring the axle to fully support the weight of the motor [11]. This structural change significantly increases the fatigue strength requirements for axles.

**Figure 2:**
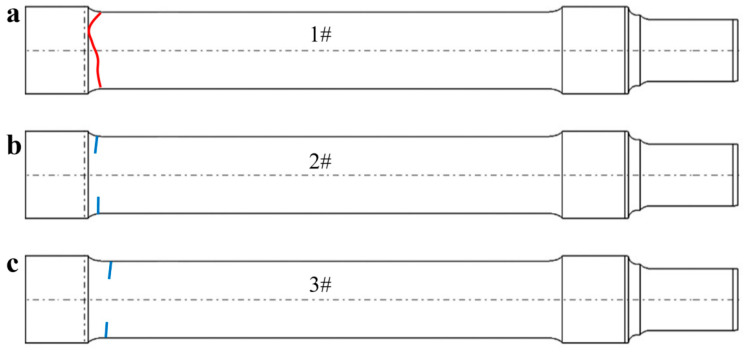
Schematic diagram of cracking locations of failure axles: (**a**) axle 1#: broken after 3 million fatigue tests, and red marked line indicates failure area; (**b**) axle 2#: cracks detected (blue line mark) after 3.5 million fatigue tests; (**c**) axle 3#: cracks detected (blue line mark) after 1.6 million fatigue tests.

During the development of a prototype PMDD electric locomotive, the newly designed axles exhibited premature failures during wheel-seat–axle fatigue testing, failing to meet validation requirements. The failed axle was made of EA1N steel, which conforms to the UIC 811-1 standard [12], and adopts the positive heat treatment process (heated to 860 ± 10 °C for 2 h followed by air cooling). Figure 2 shows the cracking locations of the three failed axles. After 3 million fatigue tests, axle 1# broke at the transition corner (marked in red). In the subsequent fatigue tests of axle 2# and axle 3#, the axle’s condition was monitored by magnetic particle inspection after stopping once a day. After 3.5 million fatigue tests, it was found that magnetic marks gathered on both sides of the transition corners (blue marks) of axle 2#. Similarly, after 1.6 million tests, accumulated magnetic marks were also found on both sides of the # 3 axle’s transition angle (marked by the blue line).

The multi-disciplinary failure analysis of three failure axes was carried out through a systematic investigation of inspection data, manufacturing processes, and operating conditions. The evaluation framework includes material quality, heat treatment parameters, machining tolerances, assembly alignment, geometric design optimization, etc., to discover the cause of insufficient fatigue life (less than the required 10^7^ cycles). The premature failure mechanisms were elucidated via surface-state-localized stress concentrations of the axle. The premature failure mechanism of the axle is elucidated based on the local stress concentration analysis of the surface state. And targeted corrective solutions were proposed.

## 2. Failure Analysis and Experimental Framework

This section provides a detailed description of the systematic investigation framework used for diagnosing the premature fatigue failure of locomotive axles. It encompasses the full suite of experimental techniques and analytical methods applied to characterize the failed component, evaluate material properties, and simulate operational stresses. Key methodologies include fractographic analysis (macro/micro), metallurgical examination, mechanical testing (tensile, impact, hardness, and fatigue), and finite element modeling (FEM) of stress distributions. The comprehensive approach ensures a thorough fundamental cause assessment of the relationship between material behavior, manufacturing characteristics, and operating load conditions.

### 2.1. Broken Axle 1# Characterization

**Figure 3:**
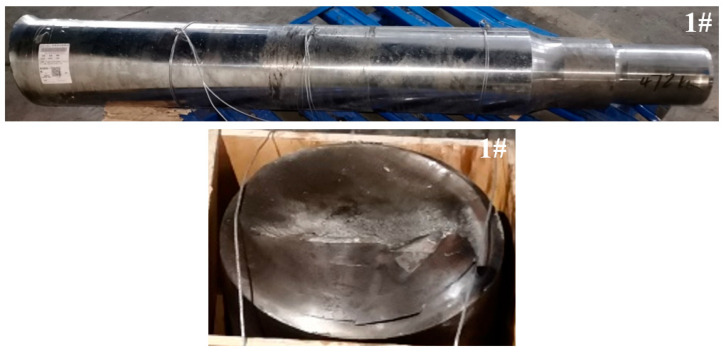
Macro-morphology of the failed axle 1# (axle diameter is 220 mm; axle shoulder diameter is 250 mm).

**Figure 4:**
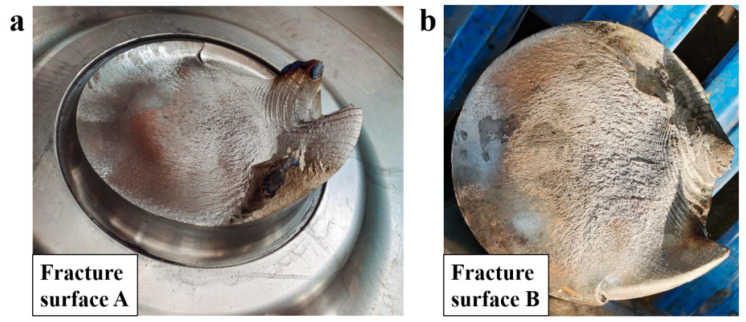
Two matching surfaces of the broken axle 1#: (**a**) surface A; (**b**) surface B.

Figure 3 shows axle #1 in its as-received condition prior to examination. The axle exhibits a complete through-thickness fracture, and the fracture occurred in the arc transitional stage. Figure 4 presents the two mating fracture surfaces of axle 1# with high morphological consistency. The fracture surfaces show metallic luster without any detectable porosity, slag inclusions, or pre-existing process-induced cracks.

**Figure 5:**
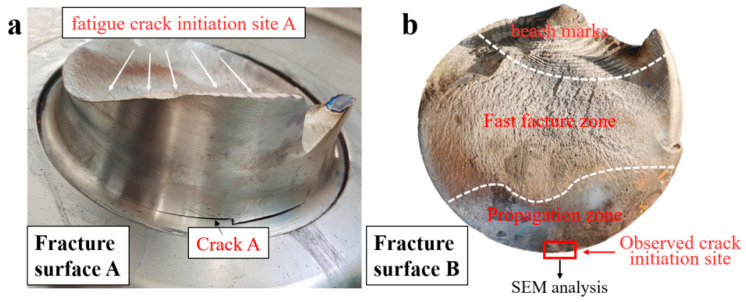
(**a**) Side view of the fracture surface A. (**b**) Fatigue propagation and fast fracture zones in fracture surface B.

As shown in Figure 5, on fracture surface A, a lightning-branched secondary crack can also be observed on the opposite side of the fracture fatigue source at fracture A. On fracture surface B, beach marks are present at both the top and bottom regions of the fracture section. The identified dual fatigue initiation sites on surfaces A and B at the transition’s rounded corners exhibit characteristic bending fatigue patterns. The fatigue propagation zone is parallel to the extrusion marks. The final fracture exhibits mixed modes: ductile shear lips at edges and cleavage-dominated brittle fracture in the central zone. The macroscopic plastic deformation is characterized by high-stress fatigue failure.

**Figure 6:**
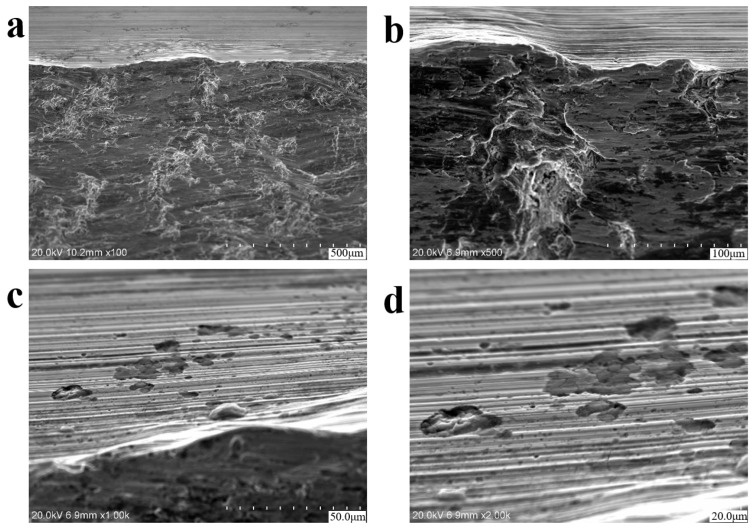
SEM morphologies near the fatigue crack initiation zone of axle 1#. (**a**,**b**) Microstructural features and fatigue striations; (**c**,**d**) parallel machining marks near the crack nucleation site.

**Figure 7:**
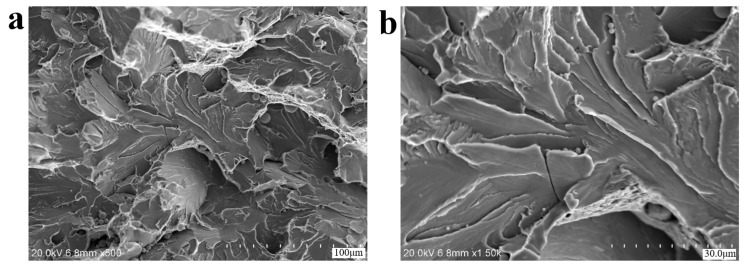
SEM morphologies of the final fracture zone of axle 1#: (**a**,**b**) cleavage facets, indicating high-stress brittle fractures.

Scanning electron microscopy (SEM) analysis specimens were extracted via wire-electrode cutting carried out along the red rectangular area marked in Figure 5b, and then, ultrasonic cleaning was carried out in anhydrous ethanol. Based on the SEM results (equipment is S-3700N Scanning Electron Microscope, Hitachi High-Tech, Tokyo, Japan), no porosity, slag inclusion, and other raw material defects were found at the crack source, and there were plastic extrusion marks on the surface. The microscopic morphology of the fracture was a quasi-cleavage fracture, as shown in Figure 6. Parallel distributions of rough machining textures can be observed on the side of the crack source, as shown in Figure 6a–d, and local corrosion pits exist. Figure 7 shows the microscopic morphology of the fast fracture zone, which is dominated by cleavage fracture and has the characteristics of brittle fractures.

### 2.2. Analyses of Unbroken Axle 2# and 3#

**Figure 8:**
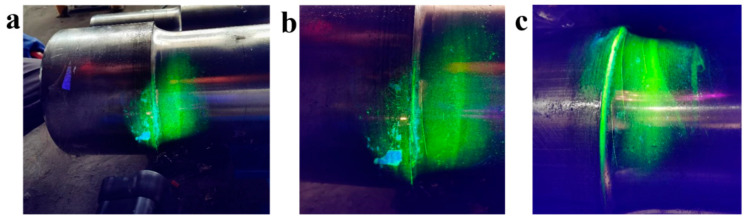
Magnetic particle detection results (**a**) showing magnetic marks distributed along the circular direction of axle 2#: (**b**,**c**) local enlarged views.

**Figure 9:**
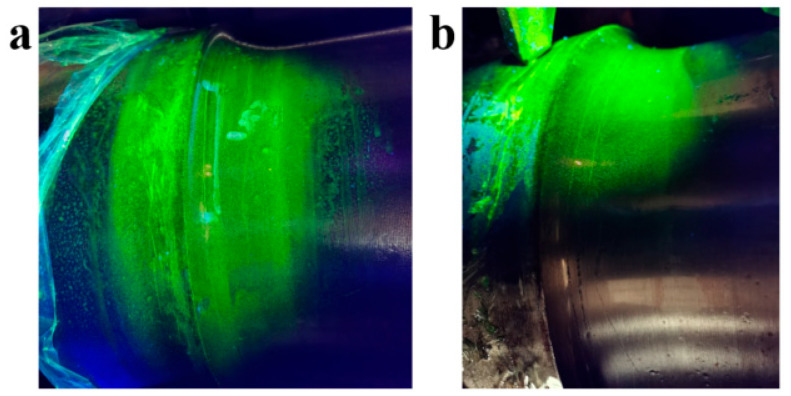
Magnetic particle detection results: (**a**) showing magnetic marks distributed along the circular direction of axle 3#, which are similar to axle 2#. (**b**) local enlarged view.

Magnetic particle detection (MPD) was used to characterize the defect locations (transition arc of the axle shoulder) of axle 2# and axle 3#. Figure 8 shows the MPD morphologies of axle 2#. There are multiple linear magnetic marks distributed along the circular direction of the axle, and the aggregation lines are distributed on both sides of the transition zone. Figure 9 shows the MPD detection morphologies of axle 3#, and the morphology and distribution of magnetic marks are similar to those of axle 2#.

**Figure 10:**
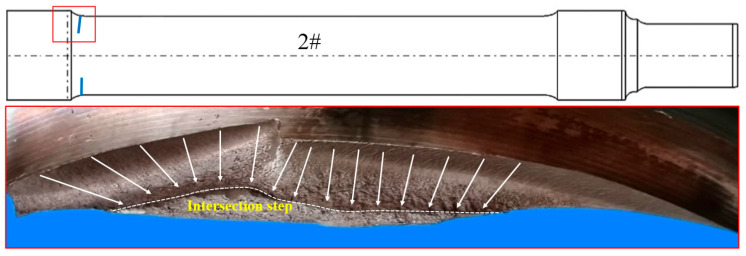
The appearance of a manually broken part at the crack initiation site of axle 2#.

**Figure 11:**
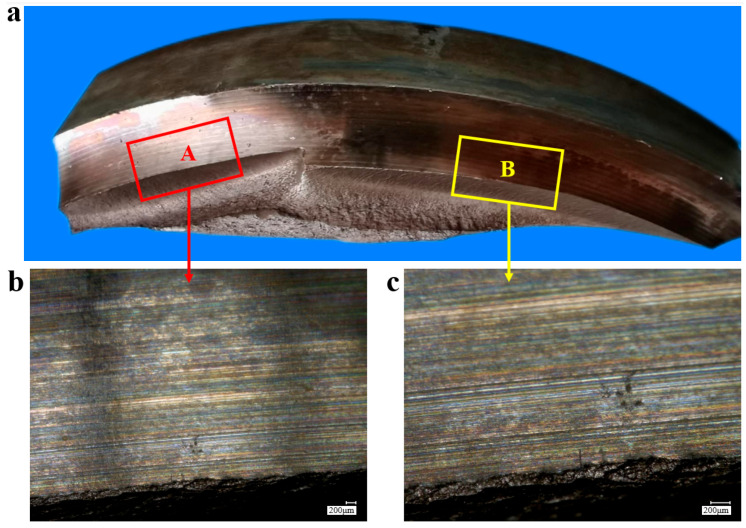
(**a**) Overall and (**b**,**c**) enlarged views of fracture sources A and B of axle 2#.

In order to obtain more information from inside the crack initiation site, axle 2# was artificially broken (firstly performed by wire cutting and then stretched and broken by a 200-ton tensile machine) along the magnetic trace, and the fracture morphology was obtained, as shown in Figure 10. There are two fracture sources in the fracture section. Fracture sources A and B expanded inward and converged to form an intersection step. Figure 11 shows a macro-diagram of the manual breaking position of axle 2#. There are parallel processing tool marks at the transition zone, as shown in Figure 11a. Figure 11b,c show the locally enlarged photos near the crack initiation site of region A and region B, respectively. Rough manufacturing marks can be observed in the figures.

**Figure 12:**
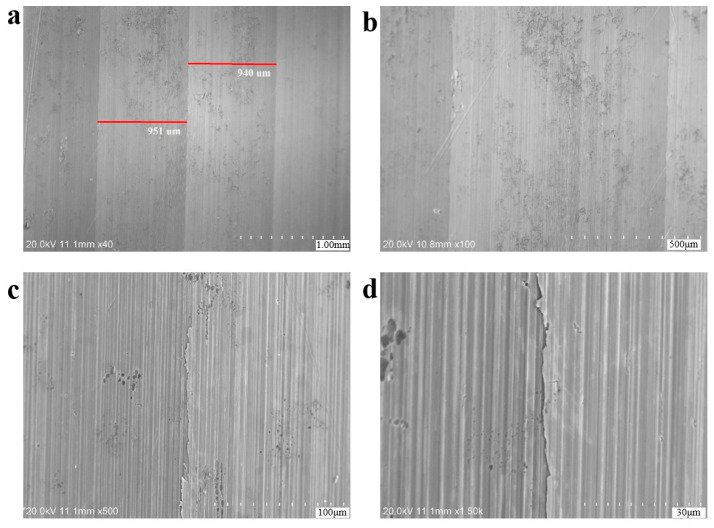
SEM morphologies of the arc transition region in axle 2# (side view): (**a**,**b**) showing microscopic morphology of the machining marks. (**c**,**d**) enlarged views.

**Figure 13:**
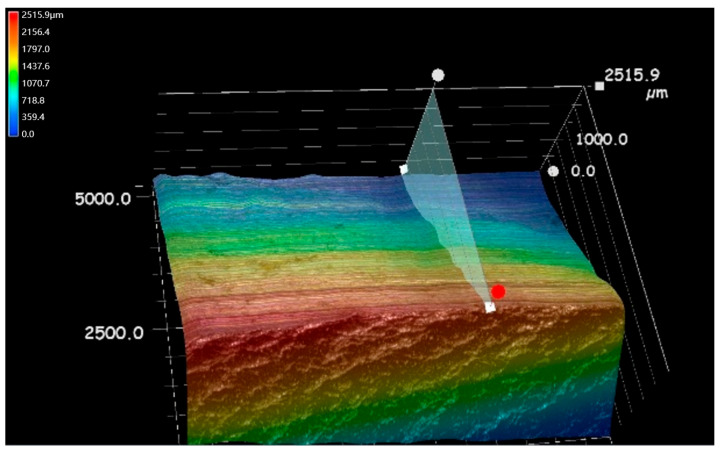
Three-dimensional morphology of the side view of region B in axle 2#.

From the SEM observations (Figure 12), it can be seen that the width of the blade marks on the surface is about 0.95 mm, and there are parallel processing textures at the bottom. As shown in Figure 13, three-dimensional confocal detection (equipment isVHX-700FC Ultra-Depth Three-Dimensional Microscope, Keyence Corporation, Osaka, Japan) was carried out on the side of region B. The height difference between the highest point and the lowest point near the transition zone is 342 μm.

**Figure 14:**
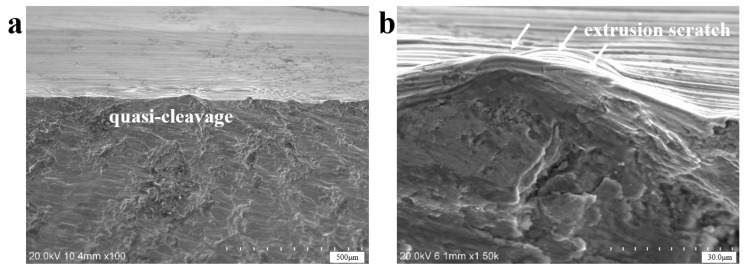
SEM morphologies of the region at the crack initiation site of axle 2#: (**a**) overall view (**b**) enlarged view of extrusion scratch.

Figure 14 shows the microscopic morphology at the crack initiation site of axle 2#. Similarly to the situation with axle 1#, there are no abnormal material defects such as porosity and slag inclusion at the crack source, and there are extrusion plastic extrusion marks at the edge. The microscopic morphology is dominated by quasi-cleavage fractures, with local extrusion scratches.

### 2.3. Chemical Composition and Metallographic Analysis

The chemical composition of axle 1#’s and axle 2#’s matrices was checked by an Optical Emission Spectrometer (equipment is SPECTRO MAXx, Kleve, Germany), and the results are shown in Table 1. The chemical composition test results are in line with the standard requirements of EA1N in UIC 811-1 [12].

**Figure 15:**
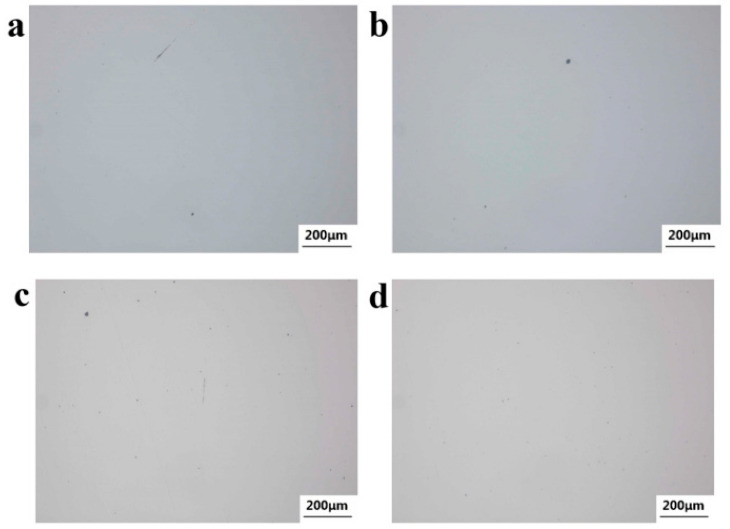
Nonmetallic inclusions distribution: (**a**,**b**) axle 1# and (**c**,**d**) axle 2#.

The metallurgical structure of the specimen was observed through the Observer A1m Metallurgical Microscope. Figure 15 shows the micro-morphology of non-metallic inclusions at 1/2R of the wheelbase surface of axles 1# and 2#. Figure 15a,b show the micro-morphology of the non-metallic inclusions of axle 1#. According to ISO 4967:2013 and GB/T 10561-2023 [13,14], their purity can reach A0.5 and DH0.5, with good purity. Figure 15c,d show the microscopic morphology of the non-metallic inclusions of axle 2#, which can be rated as A1.0 and DH0.5, and the purity is good.

**Figure 16:**
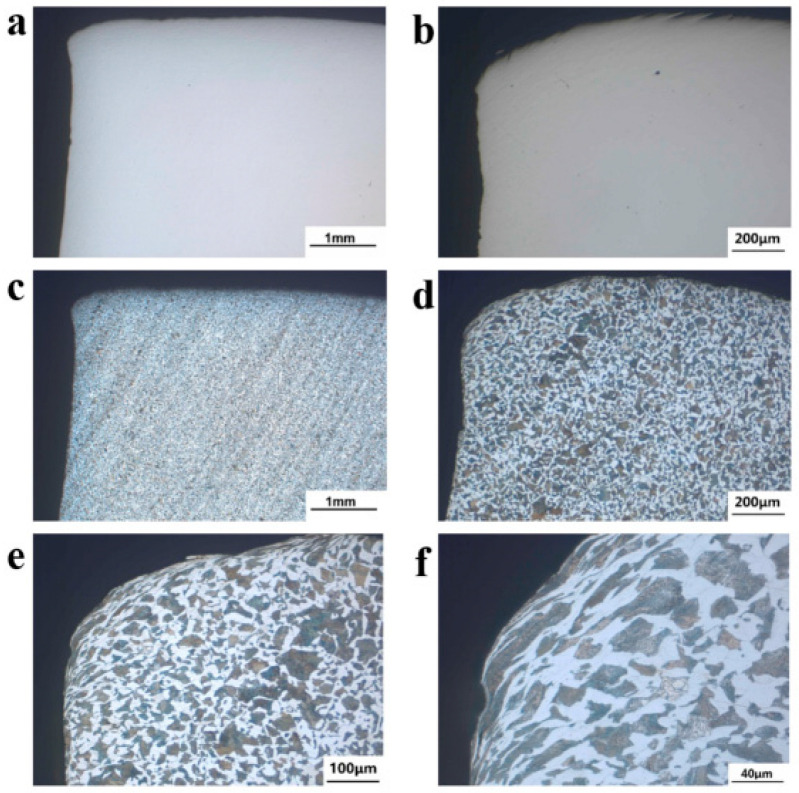
Micro-morphology and metallographic structure of the cross-section of axle 1#’s fracture source: (**a**,**b**) uneroded morphology and (**c**–**f**) metallographic structure.

In Figure 16a,b, the microscopic morphology of the fracture source section of axle 1# is shown. No defects, such as porosity, slag inclusion, and repair welding, were found in this area. After being eroded by a 2% nitrate ethanol solution, the resulting microstructure is shown in Figure 16c–f. The microstructures at the crack source are the same as those at the core, all of which are flake pearlite + ferrite. The grain size is grade 7 according to GB/T 6394-2017 and ISO 643: 2019 [15,16], and no characteristics of oxidation, decarburization, and overheated microstructure are found. The plastic extrusion traces and shear deformations observed in Figure 16e,f confirm localized yielding under high cyclic stresses. These deformations act as stress concentrators, accelerating crack nucleation.

**Figure 17:**
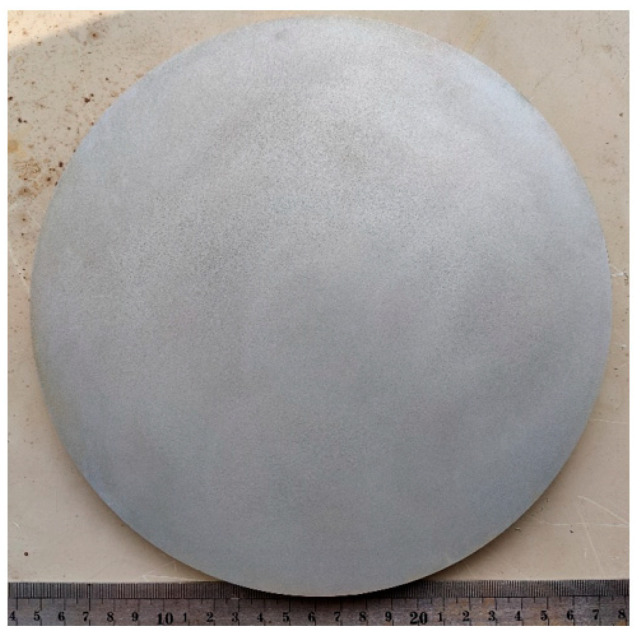
Morphology of the cross-section of axle 2#.

For further analysis, the cross-section of axle 2# was prepared as a low-power sample, and the morphology after thermal erosion using a hydrochloric acid aqueous solution is shown in Figure 17. Table 2 shows the determined results according to GB/T 1979-2001 [17], and no abnormal material defects were found.

**Figure 18:**
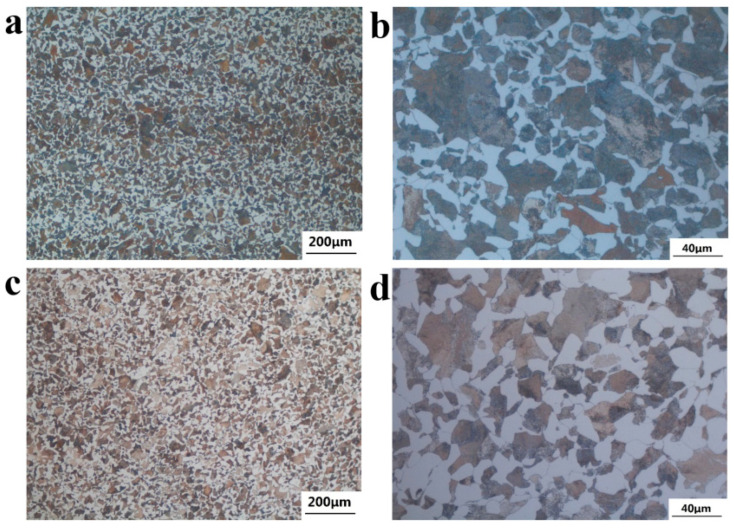
Microstructure of the substrate at 1/2R: (**a**,**b**) axle 1# and (**c**,**d**) axle 2#.

**Figure 19:**
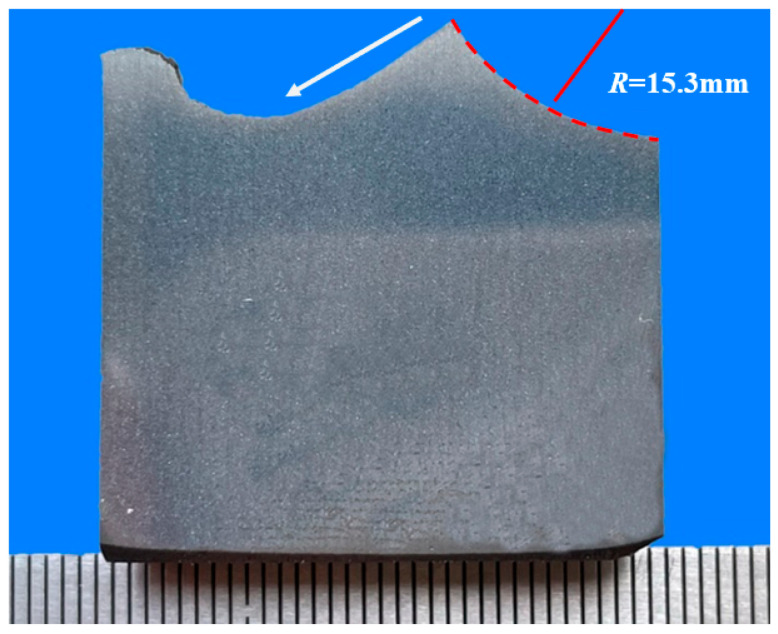
Macro-erosion morphology of the cross-section of axle 2#.

Figure 18a,b show the microstructure of the substrate at 1/2R of the surface of axle 1#. The microstructure is lamellar pearlite + ferrite, and the grain size level can be assessed as grade 7 [15,16]. Figure 18c,d show photos of the substrate’s microstructure at 1/2R of the wheelbase surface of axle 2#. The microstructure is lamellated pearlite + ferrite. Similarly to axle 1#, the grain size grade is assessed as 7. The microstructure of the transition zone was also analyzed. Figure 19 shows the low-magnification topography of the metallographic sample extracted from the crack initiation site of axle 2#. The radius of the transition zone near the fracture source is about 15.3 mm.

**Figure 20:**
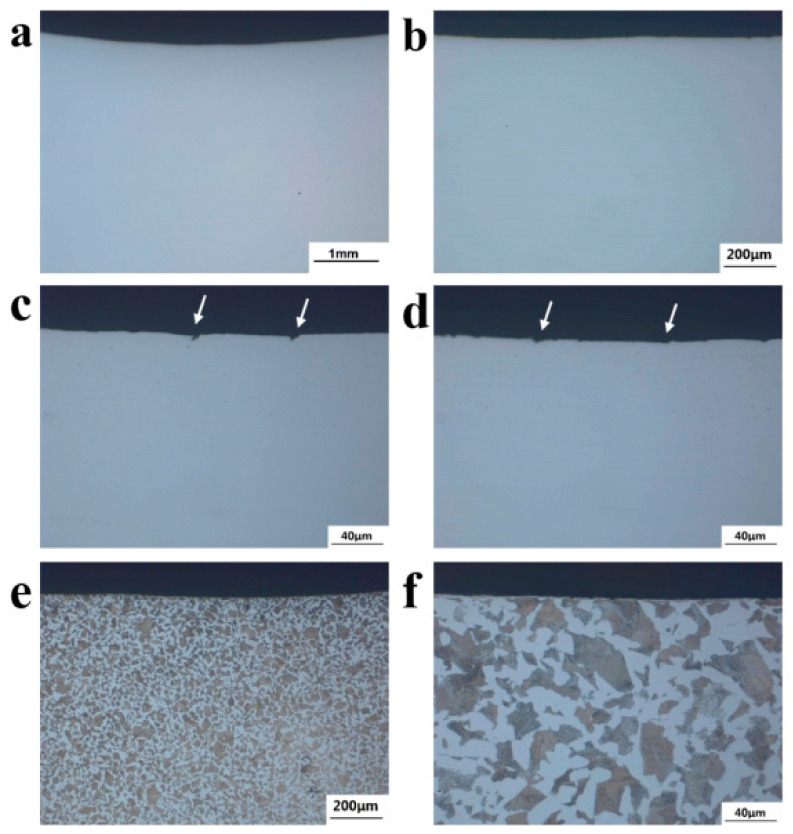
Micro-morphology of the cross-section of axle 2#’s transition zone: (**a**–**d**) uneroded morphology (there are small V-shaped notches at the section, as marked by arrows in (**c**,**d**)); (**e**,**f**) metallographic structure.

In Figure 20a–d, the microscopic morphology of the transition zone near the crack source of axle 2# is shown. There are small V-shaped notches in this section, as shown by the white arrows in Figure 20b,c. Figure 20e,f show the sectional metallographic structure of the region, and the structure is consistent with the core, which is lamellar pearlite + ferrite, with no oxidation decarburization and overheating phenomena.

**Figure 21:**
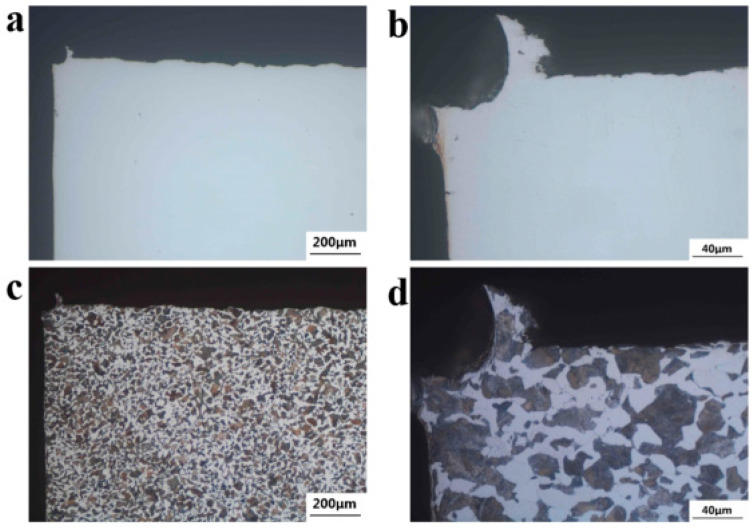
Micro-morphology near crack source section of axle 2#: (**a**,**b**) uneroded morphology and (**c**,**d**) metallographic structure.

Figure 21a,b show the microscopic morphology near the crack source section of axle 2#. There are traces of extrusion deformation at the edges, and no defects such as porosity, slag inclusion, or repair welding are found. Figure 21c,d show the sectional metallographic structure, which is consistent with the core region, and it comprises lamellar pearlite + ferrite, without oxidation, decarburization, and overheating.

### 2.4. Mechanical Property

**Figure 22:**
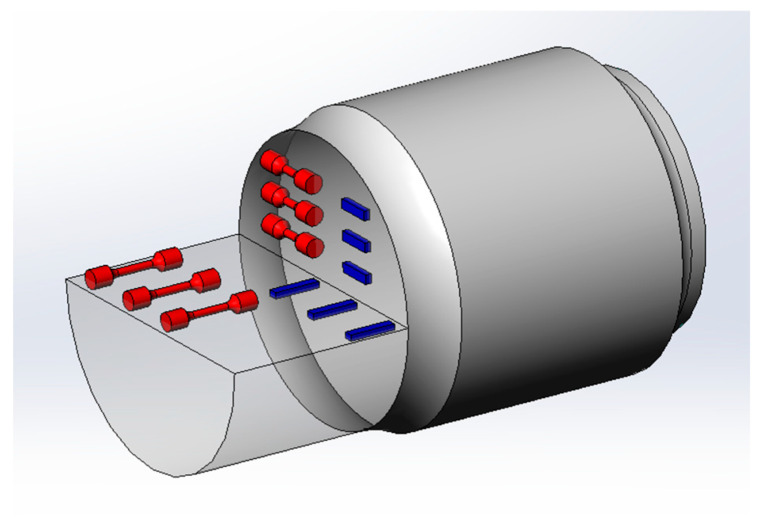
Diagram of sampling locations of the tensile and impact specimens.

The tensile and impact specimens were, respectively, taken from axles 1# and 2#, and the sampling locations are the surface, 1/2R away from the surface, and the center, as shown in Figure 22. The tested tensile properties are shown in Table 3, and all parameters meet the technical requirements of the product. The sampling direction, notch shape, test KV2 temperature, and impact absorption energy values are shown in Table 4, where the KV2 values of −20 °C at the center position are slightly lower than the technical requirements, while the rest of the testing results meet the requirements (equipment label is CMT5205 Universal Testing Machine (Shenzhen SANS, Shenzhen, China) and ZBC2302-B Pendulum Impact Tester (MTS Systems, Eden Prairie, MN, USA).

### 2.5. Fatigue Property

**Figure 23:**
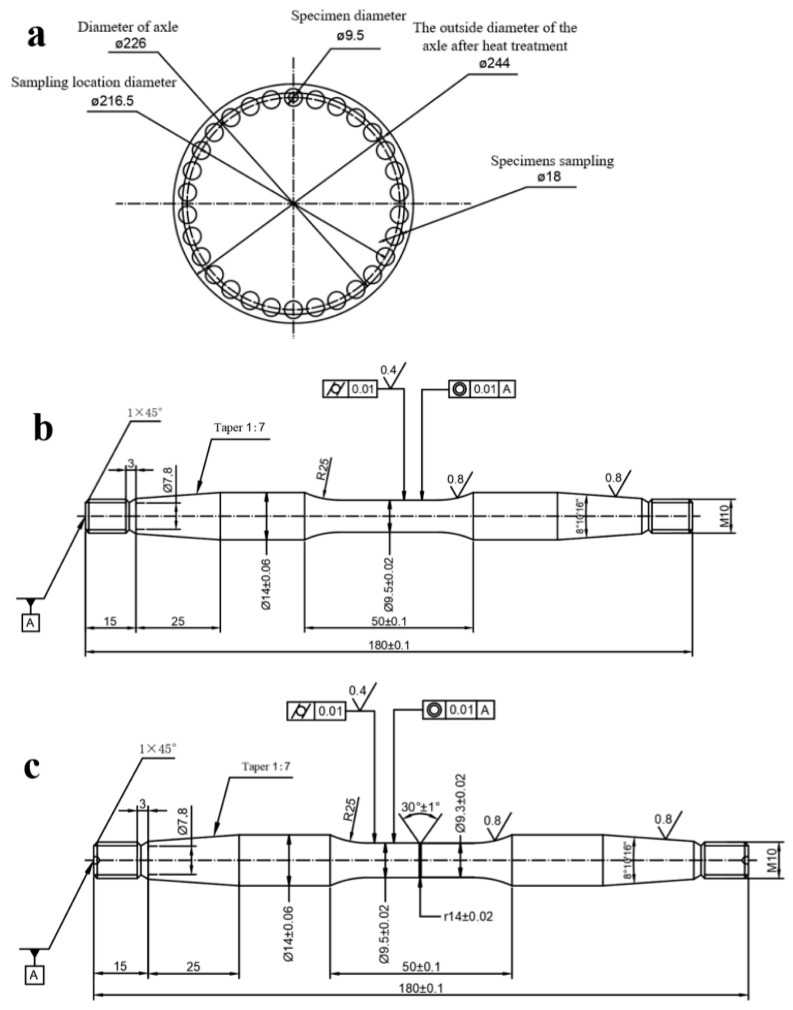
(**a**) Sampling locations and (**b**) smooth and (**c**) notched fatigue specimens.

The rotating bending fatigue test (equipment is QBWP Series Rotary Bending Fatigue Tester, Changchun Institute of Mechanical Science, Changchun, China) was carried out on the surface of the axle along the axial direction, according to the requirements of EA1N materials in EN13261 [1]. The total number of specimens is 40, and the sample shapes are smooth, cylindrical, and notched. The sampling locations and sample sizes are shown in Figure 23.

**Figure 24:**
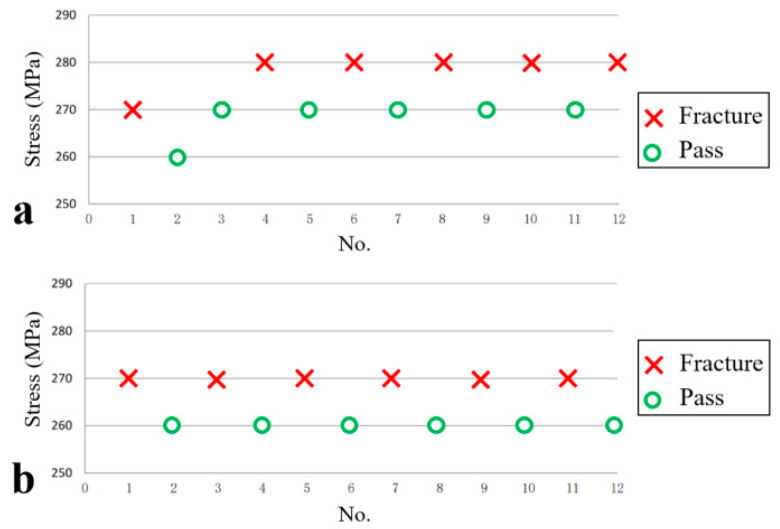
Fatigue rise and fall diagram tested at room temperature: (**a**) smooth sample and (**b**) notched sample.

The rotary bending fatigue tests were conducted according to the following procedures: (i) The actual load was calculated based on the selected stress. (ii) Weights were placed on the hanging plate, and the loading handle was rotated counterclockwise until the pointer aligned with the engraving line. (iii) The counting button was pressed to initiate the test cycle. After the specimen’s installation, alternating loads were applied until either fractures occurred or the specified cycle count was completed. Specimens fracturing outside the gauge length were considered invalid. By sorting the test data, the fatigue limit is calculated, as shown in Figure 24. Figure 24a shows the fatigue limit test data of a smooth sample, for which its fatigue limit value is 273 MPa. Figure 24b shows the fatigue limit test data of the notched samples, for which their fatigue limit value is 265 MPa. The effective stress concentration coefficient *K* of the smooth and notched fatigue specimens can be calculated as 1.03 based on the ratio of fatigue limits.

### 2.6. Residual Stress

Residual stress will exist in the process of axle surface machining, and residual stress has a great impact on the service performance of a material, such as corrosion resistance, wear resistance, and fatigue resistance. Therefore, it is necessary to detect the residual stress of an axle. A residual stress analyzer (LXRD) was used, and the parameters were as follows: sin2ψ method, Cr target, V filter, and a-Fe (211) diffraction crystal surface diffraction front (156.4°) (equipment label is X350A Residual Stress Tester, Hamamatsu, Japan). The instrument was calibrated before the test, and the zero-stress standard sample test result was 2 MPa. Three locations (axle shoulder, arc transition area of the wheel seat, and the other side of the axle shoulder) were selected to conduct the residual stress test of the axle. Four test points were applied at one location, and the tests were performed both in the X direction (circumferential) and the Y direction (axial). The residual stress test results of the axle are shown in Table 5. The surface of the arc region is manifested as tensile stress in both circumferential and axial directions. The arc transition zone exhibits residual tensile stress values of 72–99 MPa in the circumferential direction and 71–92 MPa in the axial direction. These tensile stresses, when combined with cyclic stresses during operation, directly lower the fatigue strength and accelerate the formation of cracks.

## 3. Finite Element Analysis and Fatigue Life Prediction of Axle Failures

This section employs computational methods to simulate stress distributions, predict fatigue life, and validate findings against experimental failure data. These analyses identify stress concentrations at the wheel-seat transition arc and validate the premature failure mechanisms that are observed experimentally.

### 3.1. Finite Element Model and Boundary Condition

**Figure 25:**
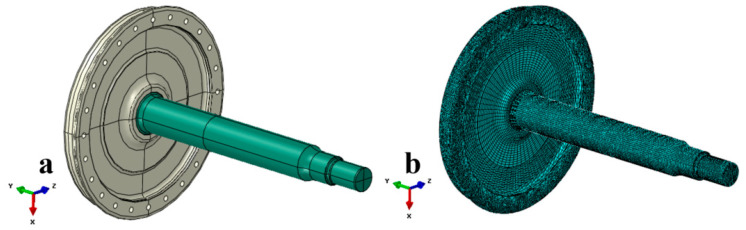
Three-dimensional model and mesh details of axle–wheel (**a**) geometry of the axle–wheel assembly model and (**b**) finite element mesh details.

Finite element analysis is carried out (using Abaqus software 2020) to further analyze whether the load applied in the fatigue-type test is consistent with the fatigue strength requirements of the axle’s design. The three-dimensional model and mesh details for axle fatigue modeling are shown in Figure 25. The axle’s material is EA1N [1,2,18], and the specific material parameters are shown in Table 6. The axle and wheel are fitted with an interference fit, and the radial interference is in the range of 0.353–0.417 mm. In order to facilitate calculations, the model is simplified, and characteristics such as pinhole chamfer are ignored on the premise of accurately reflecting the structural characteristics (as shown in Figure 25a). The analysis step is static and general, and the element type is C3D8R, containing 145,310 nodes and 197,816 elements (as shown in Figure 25b).

**Figure 26:**
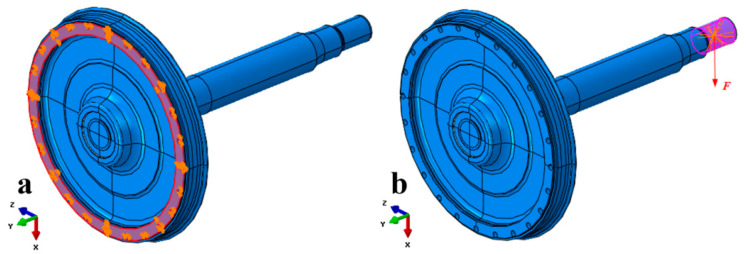
Boundary conditions of the finite element model: (**a**) fixed constraint at test bench flange; (**b**) applied actuator load and gravitational field to simulate dynamic wheel-seat bending.

In the model, a mass point is set at the axle journal to simulate the mass of the bearings, axle boxes, and loading devices. The mass point is 747 kg (7321 N), and the mass point is connected to the journal via continuous coupling. At the same time, a gravitational field is applied to the whole model. The gravitational field is oriented along the positive direction of the *X*-axis (i.e., down the vertical axis), with a gravitational acceleration of 9.8 m/s^2^. The surface-to-surface contact with friction (μ = 0.2) was used in the wheel–axle Interface. The contact surface between the wheel and the test bench’s flange is fixed, as shown in Figure 26a. We applied an actuator load of 120,361 N in the positive direction of the *X*-axis to the journal, as shown in Figure 26b. The interference contact connection between the wheel and the axle was used to calculate three working conditions: zero interference (0 mm), minimum interference amount (0.353 mm), and maximum interference amount (0.417 mm).

### 3.2. FEM Results

**Figure 27:**
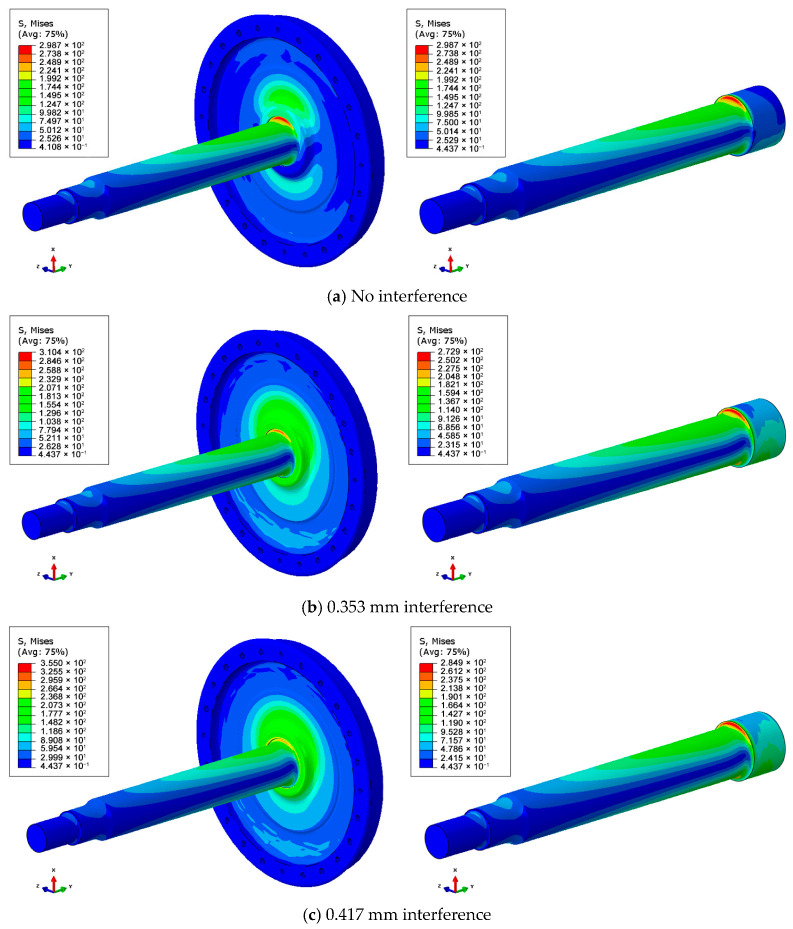
Contour plots of equivalent stress under different interference conditions: (**a**) no interference, (**b**) 0.353 mm interference, and (**c**) 0.417 mm interference.

Figure 27 shows contour plots of equivalent stresses under different conditions: without interference, 0.353 mm; with interference, 0.417 mm. The maximum equivalent stress values at the arc of the axle and wheel seat are 298.7 MPa, 272.8 MPa, and 284.8 MPa, respectively, and the maximum equivalent stress occurs at the bending compression side of the axle. The calculation results of the equivalent stress at the arc of the axle–wheel seat under the three working conditions are all greater than the 200 MPa required by the fatigue strength test.

**Figure 28:**
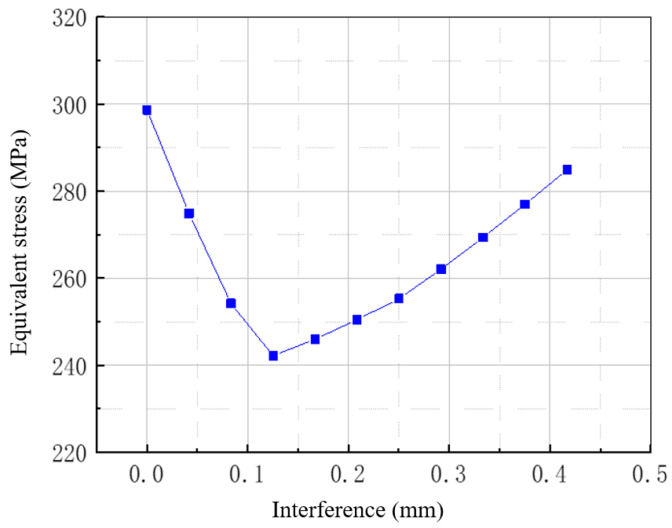
Relationship between the maximum equivalent stress and interference at the arc of the axle and wheel seat.

**Figure 29:**
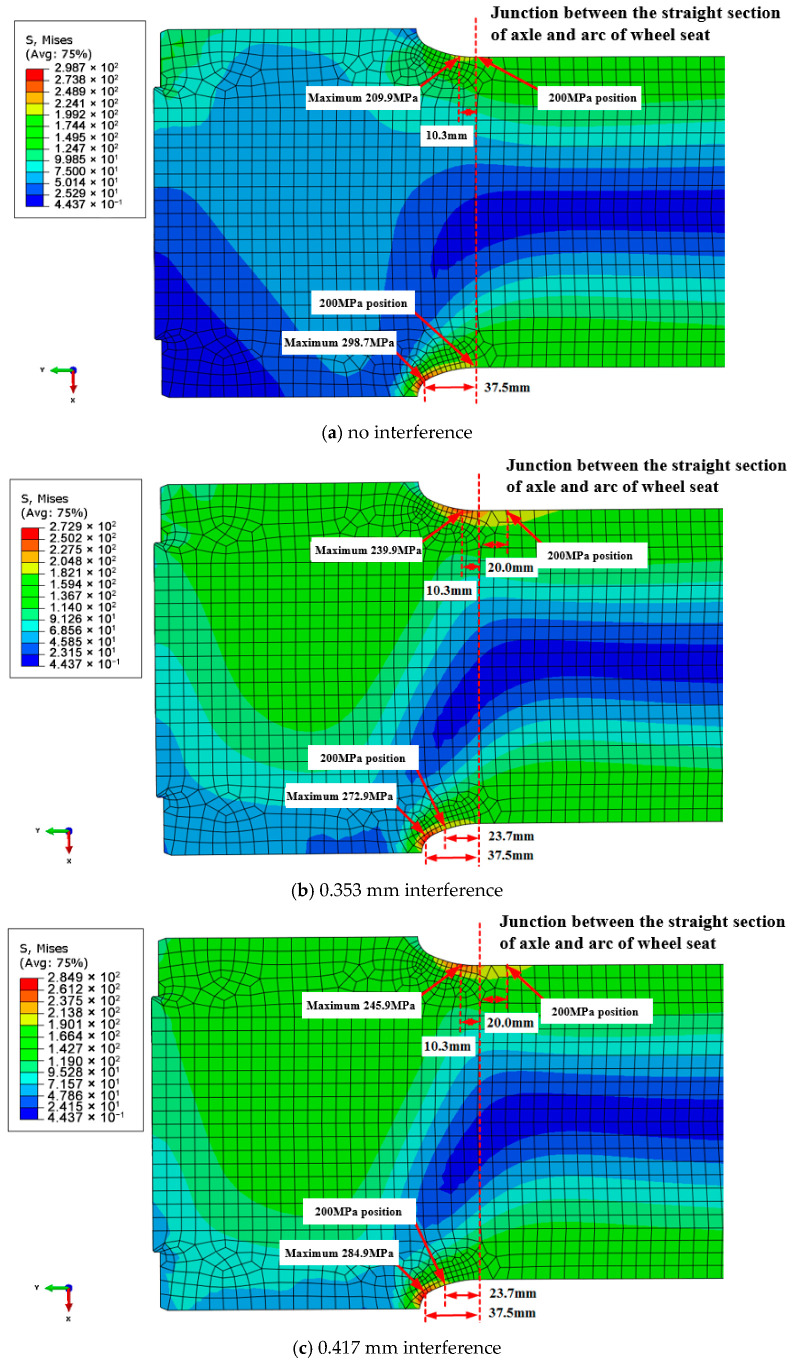
Contour plots of the local stress distribution at the junction between the straight section of the axle and arc of the wheel seat under different interference conditions: (**a**) no interference, (**b**) 0.353 mm interference, and (**c**) 0.417 mm interference.

It should be pointed out here that with an increase in the compression interference, the maximum value of the equivalent stress at the arc of the axle and wheel seat decreases first and then increases, and the change curve is shown in Figure 28. Moreover, the local stress distribution is shown in Figure 29. The maximum value and position of the equivalent stress in the axle section can be seen. In the absence of interference, the equivalent stress of 200 MPa occurs just at the junction between the flat section of the axle body and the circular arc of the axle. When interference occurs, the equivalent stress at the junction between the flat section of the axle and the circular arc of the wheel increases, and the equivalent stress of 200 MPa moves to the side of the flat section of the axle body, indicating that the interference fit alters the stress concentration zones. Under zero-interference conditions, the maximum equivalent stress (298.7 MPa) occurs at the transition arc between the wheel seat and axle body, which aligns with the crack initiation locations observed in experiments. When a 0.353 mm interference is applied, the compressive stress generated by the interference fit redistributes the load, reducing the maximum equivalent stress to 272.8 MPa. With 0.417 mm interference, the stress slightly increases to 284.8 MPa. This series of observations demonstrates a nonlinear relationship between interference magnitude and stress distribution. The predicted stress exceeds the material’s fatigue limits established in prior fatigue testing, aligning with the observed premature failure behavior.

**Figure 30:**
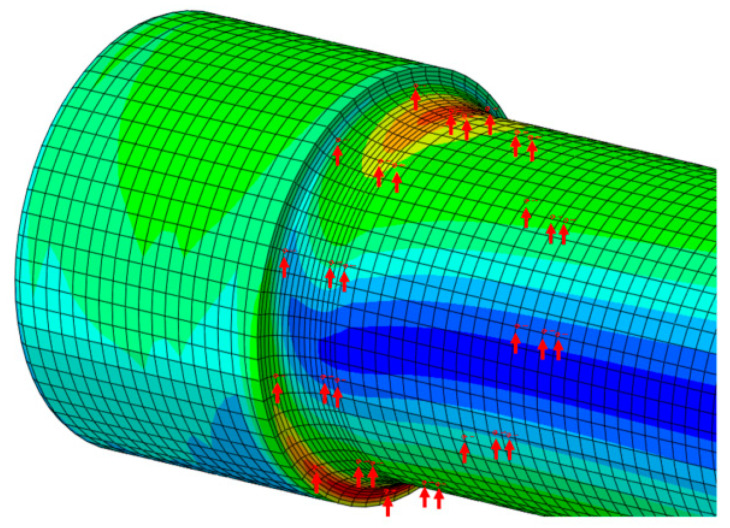
Diagram of fatigue strength evaluation nodes (marked with red arrows).

**Figure 31:**
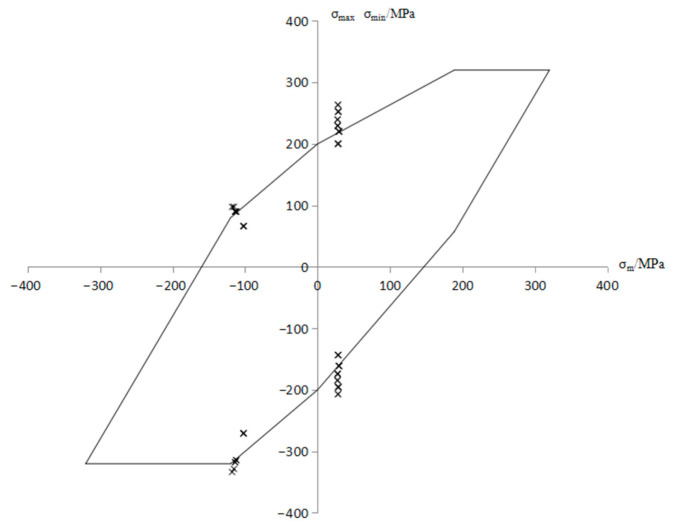
Fatigue evaluation diagram based on the Goodman diagram of each axle node.

In order to carry out fatigue strength analyses, the calculated gravity field and the actuator load are added to the negative direction of the x axis, the positive direction of the z axis, and the negative direction of the z axis to simulate the stress situation of the axis at every moment during the rotation process. The interference in the calculation is 0.417 mm. According to the above static strength analysis results, 30 nodes along the axial plane were selected as analysis objects at the junction of the plane section of the axle’s body and the arc of the wheel seat and at distances of 10.3 mm and 37.5 mm from the junction, as shown in Figure 30. Table 7 lists the average stress and stress amplitude of the 30 nodes. The average stress and maximum and minimum stress points were input into a Goodman diagram for fatigue strength evaluation [19,20]. Figure 31 shows the fatigue strength evaluation results of each node. Some nodes in the figure are outside the Goodman fatigue limit of the material, so the axle cannot meet the fatigue strength requirements under the above calculated working conditions.

The finite element results indicate that the maximum equivalent stress at the transition arc exceeds the material’s fatigue limit (200 MPa), with values ranging from 272.8 to 298.7 MPa under different interference conditions. This stress concentration is primarily attributed to the geometric discontinuity at the wheel-seat to axle-body transition. The transition arc region where the failure occurred is a critical stress concentration zone characterized by its fillet geometry. For example, the reported study by Banuta & Tarquini [9], indicated that a drive shaft failure originated at a fillet radius (1.58 mm vs. design-specified 2.5 mm), elevating local stress beyond design calculations. The geometric discontinuities (e.g., fillets, grooves) are critical stress concentrators [8]. The stress concentration factor $K_{\sigma }$ for this fillet can be estimated using the following empirical formula based on the dimensions of the axle and the fillet radius (r):

(1) $$K_{\sigma }=1+2\sqrt{\frac{t}{r}}$$

where t is the thickness of the axle at the transition zone, and r is the transition radius.

To mitigate this issue, increasing the fillet radius r is a proven approach to reducing $K_{\sigma }$. Combined with the residual tensile stress and surface roughness, the stress concentration effect becomes a dominant driver of premature crack initiation. This aligns with the Goodman diagram’s results, where nodes near the transition arc fall outside the safe fatigue limit. The FEA and stress concentration analysis jointly suggest that optimizing the transition geometry (e.g., larger fillet radius) and improving surface finish could shift the critical stress zone away from the arc, thereby extending the fatigue life.

## 4. Discussion

The fatigue limit of parts and structures is affected by the structure, size, surface, etc. The fatigue limit of structures is generally lower than the material fatigue limit of standard specimens. In the stage of axle design, because it is impossible to carry out fatigue tests on parts, the performance data of standard specimens are used as material parameters, and the fatigue limit of materials is corrected by the corresponding coefficients [21,22,23]:

(2) $$\sigma_{-1c}=\frac{\sigma_{-1}\beta_{q}}{K_{\sigma c}}$$

where $\sigma_{-1c}$ is the fatigue limit of parts; $\sigma_{-1}$ is the fatigue limit of the standard specimen; $\beta_{q}$ is the strengthening factor; $K_{\sigma c}$ is the comprehensive correction factor, which is defined as follows:

(3) $$K_{\sigma c}=\frac{K_{\sigma }}{\varepsilon_{\sigma }}+\frac{1}{\beta }-1$$

where $K_{\sigma }$ is the stress concentration factor; $\varepsilon_{\sigma }$ is the size factor; β is the surface state factor.

During operations, an interference connection is used between the axle and the wheel, the axle and the drive gear, the axle and the brake disc, and the axle and the rolling bearing. The stress state of these mating parts is far more complicated than the factors considered in axle designs. At present, many studies have shown that the relative motion between the inner hole of the wheel and the clamping seat is the main cause of the fretting fatigue of railway axles [24,25,26]. Due to the stress concentration of fretting wear and cross-sectional changes, the fatigue strength of the axle fitting area and brake disc fitting area is lower than the flat part of the axle, which is the main fatigue fracture area of the axle.

In order to improve the fatigue strength of the axle, different design ideas are adopted between the axle and the mating area between the brake disc and the axle. One is to use a large transition arc and a small diameter ratio, the main purpose of which is to reduce the stress concentration in the transition arc area, transfer the fracture danger area to the axle press assembly part, and then improve the residual stress on the wheel seat’s surface through nitriding or high-frequency quenching processes. The other idea, on the contrary, stipulates that a small transition arc and a large diameter ratio are used to reduce fretting wear in the press assembly part. The advantage of this design is that the vulnerable area is easy to monitor and problems are easy to find, but the transition arc area is prone to fatigue fracture due to stress concentration [27,28].

The three axes studied in this paper all exhibit fracture failures in the arc transition region. Considering the influence of the stress concentration, size factor, and surface factor of the axle, the fatigue strength of the material may be lower than the requirements. On the other hand, the residual tensile stress on the surface of the material is about 100 MPa, which promotes the initiation of fatigue cracks. Therefore, the rolling process of the arc section is proposed to improve the residual compressive stress of the axle transition angle. It is also recommended to optimize normalizing process parameters and cooling methods to improve the material’s strength. The newly manufactured axles did not fail prematurely in further testing.

## 5. Conclusions and Recommendations

This comprehensive investigation identifies high-stress fatigue fracture as the fundamental failure mechanism in EA1N steel axles under rotating bending loads. The key conclusions are as follows:

(1) Fractures were consistently initiated at the wheel-seat transition arc due to multi-origin fatigue crack nucleation, which has the characteristics of bidirectional rotating bending fatigue fractures.

(2) The stress of the failed axle during the fatigue test exceeds the fatigue limit of the material, which is the main reason for the insufficient fatigue life. Finite element analysis confirms local stresses exceeding 265 MPa at the transition arc, surpassing the material’s fatigue limit and explaining the premature failures occurring at 1.6–3.5 million cycles.

(3) The residual tensile stress value on the surface of the transition corner stress concentration area of the failed axle is higher, which promotes the initiation and propagation of fatigue cracks.

(4) The surface processing of the failed axle is rough, which further increases the stress concentration coefficient at the transition corner. This is a secondary cause of insufficient fatigue life.

(5) We recommend changing the surface machining method to using rolling technology in order to improve the residual compressive stress in the axle transition zone. The normalizing process parameters and heat treatment cooling methods can be optimized to further reduce the residual tensile stress on the axle’s surface.

## Figures and Tables

**Table 1:** Chemical composition of axles 1# and 2# (wt%).

Element	C	Si	Mn	P	S	Cr	Ni	Mo	Cu	V	Al
1#-Axle	0.38	0.31	1.00	0.016	0.002	0.11	<0.01	<0.01	<0.01	<0.01	0.03
2#-Axle	0.33	0.30	0.97	0.012	0.002	0.09	<0.01	0.01	0.01	<0.01	0.03
Standard value	≤0.37	≤0.46	≤1.12	≤0.040	≤0.040	≤0.30	≤0.30	≤0.05	≤0.30	≤0.05	-

**Table 2:** Low-power rating results of axle 2#.

No.	Center Loose	Generally Loose	Ingot Segregation	Spot Segregation
Axle 2#	0.5	0.5	Not observed	Not observed

**Table 3:** Results of tensile properties of axles 1# and 2#.

Specimen No.	*R*e (MPa)	*R*m (MPa)	*A* (%)
1#-surface-1	355	642	22.0
1#-surface-2	371	635	28.5
1#-surface-3	361	631	28.0
1#-1/2R-1	366	646	27.5
1#-1/2R-2	377	647	26.5
1#-1/2R-3	370	639	26.5
1#-middle-1	362	629	26.5
1#-middle-2	346	601	22.0
1#-middle-3	361	617	29.0
2#-surface-1	366	607	24.0
2#-surface-2	373	611	26.5
2#-surface-3	359	610	26.0
2#-1/2R-1	358	594	29.0
2#-1/2R-2	348	585	30.0
2#-1/2R-3	357	596	28.0
2#-middle-1	358	577	27.5
2#-middle-2	349	573	29.5
2#-middle-3	361	591	25.0
Requirements	≥320	550~650	≥22

**Table 4:** Results of impact properties of axles 1# and 2#.

Specimen No.	Direction	Temperature (°C)	KV2 (J)				
			No. 1	No. 2	No. 3	Average Value	Requirements
1#-surface	Transverse	Room temperature	81	71	80	77.3	≥25
1#-1/2R			70	67	64	67.0	
1#-middle			68	72	73	71.0	
1#-surface	Transverse	−20	22	13	29	21.3	≥10
1#-1/2R			12	17	9.6	12.9	
1#-middle			21	21	23	21.7	
1#-surface	Longitudinal		24	27	26	25.7	≥17
1#-1/2R			25	21	23	23.0	
1#-middle			12	11	26	16.3	
1#-surface	Transverse	−40	14	10	11	11.7	-
1#-1/2R			11	12	9.2	10.7	
1#-middle			14	8.4	10	10.8	
1#-surface	Longitudinal		14	15	23	17.3	
1#-1/2R			10	9.8	11	10.3	
1#-middle			16	11	16	14.3	
2#-surface	Transverse	Room temperature	75	82	70	75.7	≥25
2#-1/2R			85	85	88	86.0	
2#-middle			82	92	90	88.0	
2#-surface	Transverse	−20	20	21	18	19.7	≥10
2#-1/2R			20	22	12	18.0	
2#-middle			29	18	15	20.7	
2#-surface	Longitudinal		26	15	22	21.0	≥17
2#-1/2R			16	15	23	18.0	
2#-middle			21	11	14	15.3	
2#-surface	Transverse	−40	10	16	8.2	11.4	-
2#-1/2R			9.0	11	9.9	10.0	
2#-middle			11	11	12	11.3	
2#-surface	Longitudinal		17	7.7	15	13.2	
2#-1/2R			12	15	11	12.7	
2#-middle			6.7	9.9	9.2	8.6	

**Table 5:** Testing results of residual stress.

Test Zones	Direction	Test Values (MPa)			
		1	2	3	4
Arc transition region	Circumferential	94	99	72	82
	Axial	89	92	71	78

**Table 6:** Material parameters of EA1N steel used in modeling [1,2,18].

Parameters	Values
Modulus of elasticity (GPa)	210
Poisson’s ratio	0.3
Density (kg/m^3^)	7800
Tensile strength (MPa)	550–650
Yield strength (MPa)	≥320
Fatigue strength (MPa)	≥200

**Table 7:** Stress distribution in each node of axle.

Nodes	Average Stress (MPa)	Stress Amplitude (MPa)	Principal Stress (MPa)	
			Maximum	Minimum
40	27.68	211.95	239.63	−184.27
73	27.40	212.24	239.65	−184.84
1027	27.82	201.77	229.59	−173.95
1033	28.34	171.76	200.09	−143.42
1188	−115.37	213.16	97.79	−328.53
1196	28.21	235.32	263.52	−207.11
1417	28.40	171.82	200.21	−143.42
1423	27.75	201.86	229.60	−174.11
2014	28.4	171.74	200.17	−143.30
2020	27.77	201.90	229.67	−174.13
2476	28.31	235.24	263.55	−206.93
2484	−117.89	215.66	97.766	−333.55
2594	27.87	201.72	229.59	−173.85
2600	28.379	171.78	200.16	−143.41
11,393	28.63	223.95	252.58	−195.32
11,447	29.50	190.76	220.26	−161.25
11,510	−112.27	202.18	89.91	−314.44
11,540	−102.34	168.53	66.19	−270.86
14,512	29.61	190.85	220.45	−161.24
14,566	28.47	224.14	252.61	−195.67
14,617	−102.24	168.77	66.52	−271.01
14,647	−113.74	203.76	90.02	−317.50
18,198	−113.77	203.91	90.14	−317.68
18,228	−101.99	168.38	66.39	−270.37
18,277	28.50	224.20	252.71	−195.70
18,331	29.6749	190.71	220.38	−161.03
23,043	−102.12	168.42	66.30	−270.54
23,073	−112.10	201.98	89.87	−314.08
23,134	29.583	190.78	220.36	−161.19
23,188	28.71	223.87	252.58	−195.16

## Data Availability

The original contributions presented in this study are included in the article. Further inquiries can be directed to the corresponding author.
